# Diagnostic Accuracy of Molecular Amplification Tests for Human African Trypanosomiasis—Systematic Review

**DOI:** 10.1371/journal.pntd.0001438

**Published:** 2012-01-10

**Authors:** Claire M. Mugasa, Emily R. Adams, Kimberly R. Boer, Heleen C. Dyserinck, Philippe Büscher, Henk D. H. F. Schallig, Mariska M. G. Leeflang

**Affiliations:** 1 Royal Tropical Institute, KIT Biomedical Research, Amsterdam, The Netherlands; 2 Department of Veterinary Parasitology and Microbiology, Faculty of Veterinary Medicine, Makerere University Kampala, Kampala, Uganda; 3 Amsterdam Institute of Global Health and Development, Amsterdam, The Netherlands; 4 Academic Medical Centre Library, Academic Medical Centre, Amsterdam, The Netherlands; 5 Department of Biomedical Sciences, Institute of Tropical Medicine, Antwerp, Belgium; 6 Department of Clinical Epidemiology, Biostatistics and Bioinformatics, Academic Medical Centre, Amsterdam, The Netherlands; International Centre of Insect Physiology and Ecology, Kenya

## Abstract

**Background:**

A range of molecular amplification techniques have been developed for the diagnosis of Human African Trypanosomiasis (HAT); however, careful evaluation of these tests must precede implementation to ensure their high clinical accuracy. Here, we investigated the diagnostic accuracy of molecular amplification tests for HAT, the quality of articles and reasons for variation in accuracy.

**Methodology:**

Data from studies assessing diagnostic molecular amplification tests were extracted and pooled to calculate accuracy. Articles were included if they reported sensitivity and specificity or data whereby values could be calculated. Study quality was assessed using QUADAS and selected studies were analysed using the bivariate random effects model.

**Results:**

16 articles evaluating molecular amplification tests fulfilled the inclusion criteria: PCR (n = 12), NASBA (n = 2), LAMP (n = 1) and a study comparing PCR and NASBA (n = 1). Fourteen articles, including 19 different studies were included in the meta-analysis. Summary sensitivity for PCR on blood was 99.0% (95% CI 92.8 to 99.9) and the specificity was 97.7% (95% CI 93.0 to 99.3). Differences in study design and readout method did not significantly change estimates although use of satellite DNA as a target significantly lowers specificity. Sensitivity and specificity of PCR on CSF for staging varied from 87.6% to 100%, and 55.6% to 82.9% respectively.

**Conclusion:**

Here, PCR seems to have sufficient accuracy to replace microscopy where facilities allow, although this conclusion is based on multiple reference standards and a patient population that was not always representative. Future studies should, therefore, include patients for which PCR may become the test of choice and consider well designed diagnostic accuracy studies to provide extra evidence on the value of PCR in practice. Another use of PCR for control of disease could be to screen samples collected from rural areas and test in reference laboratories, to spot epidemics quickly and direct resources appropriately.

## Author Summary

A range of molecular amplification techniques has been developed for the diagnosis of HAT, with polymerase chain reaction (PCR) at the forefront. As laboratory strengthening in endemic areas increases, it is expected that the applicability of molecular tests will increase. However, careful evaluation of these tests against the current reference standard, microscopy, must precede implementation. Therefore, we have investigated the published diagnostic accuracy of molecular amplification tests for HAT compared to microscopy for both initial diagnosis as well as for disease staging.

Here, PCR tests seem to have an acceptably high specificity and sensitivity for diagnosis of stage I HAT. This conclusion is, however, based on multiple-microscopy based techniques as reference standards, which may have low sensitivity, and a patient population that was not always representative. Future studies should, therefore, first and foremost include those patients for which PCR may become the test of choice. More certainty about the practical value of PCR tests for HAT diagnosis should come from non-accuracy design studies, like feasibility or cost-effectiveness studies.

## Introduction

Human African trypanosomiasis (HAT), also known as sleeping sickness, is a parasitic disease caused by single-celled, eukaryotic protozoa called trypanosomes. Two subspecies of *Trypanosoma brucei* namely *T. b. gambiense* and *T. b. rhodesiense*, cause the disease in West and Central Africa and in East Africa respectively [1]. In recent years the number of HAT patients has fallen due to the renewal of control programs in the late 1990's; however the current number of patients reported for treatment per year in Africa is still approximately 10,000; with an estimated number of infected patients around three times that number [1]. The reference standard diagnostic test for HAT is microscopy, whereby demonstration of parasites in the body fluids confirms active infection [2], [3]. Microscopy is a compelling diagnostic tool due to its high specificity, ease of use, lack of cold chain, lack of electricity requirements and hence ability to be taken into rural areas where HAT is prevalent. However, its lack of sensitivity (approximately 10,000 parasites/ml for wet blood film examination) means that many patients may not be positively diagnosed (false negative) which may lead to death of patients in the absence of treatment [2]. Only with concentration methods such as microhaematocrit centrifugation [3], quantitative buffy coat technique (QBC) [4] and mini-anion-exchange centrifugation technique (mAECT) [5], [6] can microscopy detect parasitaemia as low as 50 parasites/ml. This limits the utility of microscopy in resource-poor settings, as these concentration methods require electricity and other laboratory logistics. Regardless, microscopy still remains the basis of HAT diagnosis, disease staging and after-treatment follow-up due to its high specificity and availability.

HAT comprises two stages of disease; stage one affects the blood, lymph and peripheral organs; stage two occurs when parasites enter the central nervous system. Currently, staging of HAT is performed by microscopic examination of cerebrospinal fluid (CSF) for presence of parasites and an increased white blood cell (WBC) count (WHO 1986). Patients with stage one HAT should be treated with pentamidine (*T. b. gambiense*) or suramin (*T. b. rhodesiense*) [7]. Stage two drugs must be able to cross the blood brain barrier (BBB); melarsoprol is a commonly administered drug for treatment of this stage but can cause reactive encephalopathy with sometimes fatal outcome [8]. The newly recommended treatment for stage two *T.b. gambiense* HAT, i.e. nifurtimox-eflornithine combination is less toxic but administration is still complex [9]. It is therefore, crucial to reduce false positives and, subsequently also, determine the appropriate treatment by accurate disease stage determination.

Recently, a range of molecular amplification techniques have been developed for the diagnosis of HAT, with polymerase chain reaction (PCR) at the forefront [10]–[12]. These tests are not commonly used in endemic areas due to the necessity of continuous electricity, trained staff, sophisticated equipment, and the requirement of a cold chain. Isothermal reactions such as loop-mediated isothermal amplification (LAMP) [13], and nucleic acid sequence-based amplification (NASBA) [15], [16] have also been proposed for the diagnosis of HAT. These diagnostic tests may be more applicable for HAT diagnosis because they need less expensive equipment and post-amplification handling requirements that are imposed by PCR testing. If the available molecular amplification diagnostic tests are to be safely used to support HAT diagnosis, they must have high diagnostic specificity as well as sensitivity to ensure that the dangers of inappropriate treatment are avoided.

As laboratory strengthening in endemic areas increases, it is expected that the applicability of molecular tests will increase. However, careful evaluation of these tests against the current reference standard, microscopy, must precede implementation. Therefore, we have investigated the published diagnostic accuracy of molecular amplification tests for HAT compared to microscopy for both initial diagnosis as well as for disease staging. Furthermore, we investigated reasons for variation in accuracy amongst HAT diagnostic tests.

## Materials and Methods

### Searching

In order to find all relevant articles assessing the diagnostic accuracy of molecular assays for HAT, MEDLINE and EMBASE databases were searched with a combination of the following search terms as MeSH (Medical Subject Headings) terms and/or free text words; see Appendix S1. Abstracts of study articles published until the 4^th^ March 2011 were identified electronically in Medline and Embase. Unpublished data were sought from scientific conference abstract books, symposia, books and experts (Institute of Tropical Medicine, Antwerp, Belgium; Makerere University Kampala, Uganda and Centre International de Recherche-Dévelopement sur l'Elevage en Zone Humide, Bobo Dioulasso, Burkina Faso). The reference lists of included studies and of review articles were checked to identify additional studies for inclusion.

Articles were initially screened on the title and secondly upon reading the abstract. At this stage, articles not using molecular techniques for diagnostic purposes, case-studies (only patients with confirmed HAT), review articles, serological diagnostics studies and studies only diagnosing animal trypanosomiasis or other non-HAT trypanosomes were excluded. All studies highlighted by at least one of the two review authors were selected; if either reviewer was unsure about exclusion then the article was included to the next stage. The full text of appropriate articles was read and taken forward for study selection. Study selection was conducted by two authors (CM and EA) independently, in the case of disagreements a third author (either KB or ML) acted as a mediator.

### Selection

We included all studies that evaluated the accuracy of molecular tests for either HAT, for one of the two subspecies of trypanosomes (i.e. East Africa or Central and Western Africa), or for stage two HAT. Studies were included if they involved clinical specimens of patients suspected of any form of HAT and fulfilled the following inclusion criteria:

Any study design (case-control, consecutive and cross-sectional studies), as long as the study involved human clinical samples and both diseased and non-diseased patients.
The use of the reference standard, microscopy of trypanosomes in blood or cerebrospinal fluid (CSF) or lymph node aspirate. Differentiation between different microscopic techniques was not made, although taken into account during the quality assessment of the articles.

### Data abstraction

Diagnostic accuracy data for two-by-two contingency tables, patient spectrum data and quality assessment data were extracted by two independent review authors (CM and EA) and recorded onto a standard form. Discrepancies were resolved by mediation of a third researcher (ML). From each study, the following characteristics were extracted: i) molecular test type; ii) clinical material assessed (blood, cerebrospinal fluid; iii) the sub-species detected (*T.b. gambiense* or *T.b. rhodesiense*); iv) read-out method of index test e.g. oligochromatography (OC); v) target gene of the index test; vi) study design i.e. whether the patients were equally suspected (‘consecutive design’) or if cases and controls were selected from different populations (‘case-control study’). Quality assessment was based on QUADAS (Quality Assessment of Diagnostic Accuracy Studies) [17].

### Quantitative data synthesis

The estimates of sensitivity and specificity and their 95% confidence interval were plotted in forest plots and receiver operating characteristic (ROC) space in Review Manager version 5. For the meta-analysis, we used the bivariate random effects model through Proc NLMIXED in SAS for Windows, version 9.2 (Cary, NC). This model pools sensitivity and specificity in one model, while accounting for the correlation between the two [18]. Studies that evaluated the diagnostic value of the tests were analyzed separately from studies that evaluated the staging value of the tests. Articles in which two-by-two contingency tables could not be completed were excluded from the meta-analyses.

Summary estimates of sensitivity and specificity for diagnosis and staging for the different assays were calculated. Meta-analysis was performed if at least three studies evaluated the same assay in the same sample type (either blood or CSF). Real-time assays were considered as different assays than standard assays, because of significant differences in protocol and design of primer/probe mixes.

The results in diagnostic accuracy reviews are expected to show much heterogeneity, mainly due to threshold effects. It is therefore more common to investigate the sources of heterogeneity, without formally testing whether heterogeneity is present or not [19]. For the same reasons, a standard random effects model was used. Heterogeneity was investigated by adding the following covariates to the meta-regression models, if appropriate and possible: i) type of detection system; ii) tissue used e.g. blood versus CSF; iii) sub-species detection *T.b. gambiense* or *T.b. rhodesiense*; iv) target gene of the index test; v) study design and quality indicated by consecutive versus case-control studies.

All reporting in this review is in accordance with the MOOSE guidelines [20].

## Results

### Flow of included studies

**Figure 1:**
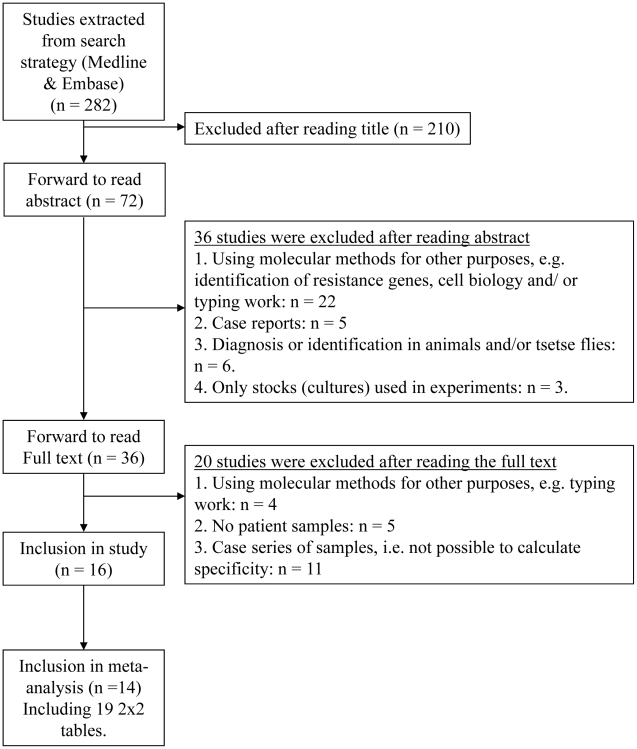
Flow of included studies.

The electronic searches yielded a total of 282 articles (see Figure 1). After reading the title and abstract, thirty-six articles were taken forward and the full text article was read. Twenty articles were excluded at this stage; 4 articles used molecular methods for other purposes e.g. genotyping data, 5 articles did not test patient samples and 11 articles reported case series where the specificity could not be calculated. Sixteen articles were selected for inclusion in the systematic review.

### Study characteristics

The index tests assessed were; PCR (n = 12) [11], [21]–[31], NASBA (n = 2) [15],[16], LAMP (n = 1) [13] and a study comparing PCR and NASBA (n = 1) [23]. Two studies assessed PCR combined with Oligochromatography (PCR-OC) and three studies assessed NASBA combined with Oligochromatography (NASBA-OC). One study [15] assessed a real-time NASBA assay (RT-NASBA).

Ten publications focused on the primary diagnosis of HAT in blood, one of these used CSF and blood for diagnosis of HAT. Two publications reported on both diagnosis and staging and used blood for diagnosis and CSF for staging. The two publications focusing only on staging both used CSF for this purpose. See Table 1 for full details.

**Table 1:** Characteristics of all papers included in the review, ordered by year.

Publication (reference)	Aim	Index test	Region	Country	Infecting Subspecies	Clinical sample	Study design
Kabiri et al., 1999 [11]	Diagnosis	PCR	CWA	Equatorial Guniea, Angola	T.b.gambiense	Blood	Consecutive
Truc et al., 1999 [24]	Staging	PCR	CWA	Cote d'Ivoire	T.b.gambiense	CSF	Consecutive
Kyambadde et al., 2000 [28]	Diagnosis	PCR	CWA	Uganda	T.b.gambiense	Blood & CSF	Consecutive
Penchenier et al., 2000 [30]	Diagnosis	PCR	CWA	Cameroon	T.b.gambiense	Blood	Consecutive
Radwanska et al., 2002 [31]	Diagnosis	PCR	CWA	Cote d'Ivoire	T.b.gambiense	Blood	Consecutive
Solano et al., 2002 [26]	Diagnosis	PCR	CWA	Central Cote d'Ivoire	T.b.gambiense	Blood	Consecutive
Jamonneau et al. 2003 [25]	Staging	PCR	CWA	Central Cote d'Ivoire	T.b.gambiense	CSF	Consecutive
Becker 2004 [32]	Development studies	RT-PCR	CWA	South Sudan	T.b.gambiense	Blood	Case series
Picozzi et al., 2005 [27]	Diagnosis	PCR	CWA & EA	South Sudan, North-West Uganda	T.b.gambiense & rhodesiense	Blood	Case control
Deborggraeve et al., 2006 [21]	Diagnosis	PCR-OC	CWA	D.R. Congo	T.b.gambiense	Blood	Case control
Koffi et al., 2006 [29]	Diagnosis	PCR	CWA	Central Cote d'Ivoire	T.b.gambiense	Blood	Consecutive
Njiru et al., 2007 [13]	Development studies	LAMP	CWA & EA	Uganda, Tanzania	T.b.gambiense & rhodesiense	Blood & CSF	Case series
Mugasa et al., 2008 [15]	Diagnosis	NASBA-RT	EA	Uganda	T.b.rhodesiense	Blood	Case Control
Mugasa et al., 2009[16]	Diagnosis and staging	NASBA-OC	CWA & EA	D.R.Congo, Uganda	T.b.gambiense & rhodesiense	Blood & CSF	Case control (blood); Consecutive (CSF)
Matovu et al., 2010 [23]	Diagnosis	PCR-OC & NASBA-OC	CWA & EA	D.R.Congo, Uganda	T.b.gambiense & rhodesiense	Blood	Case control
Deborggraeve et al., 2011 [22]	Diagnosis and staging	PCR	CWA	D.R. Congo	T.b.gambiense	CSF	Case control (blood); Consecutive (CSF)

CWA = Central and West Africa; EA = East Africa. Development study = article in which protocol is developed. PCR = Polymerase Chain Reaction; RT-PCR = Real-time PCR; PCR-OC = PCR-oligochromatography; LAMP = Loop-mediated isothermal amplification; NASBA = Nucleic acid sequence based amplification; NASBA-RT = Real-time NASBA. CSF = Central Spinal Fluid.

### Quality of study reports

**Figure 2:**
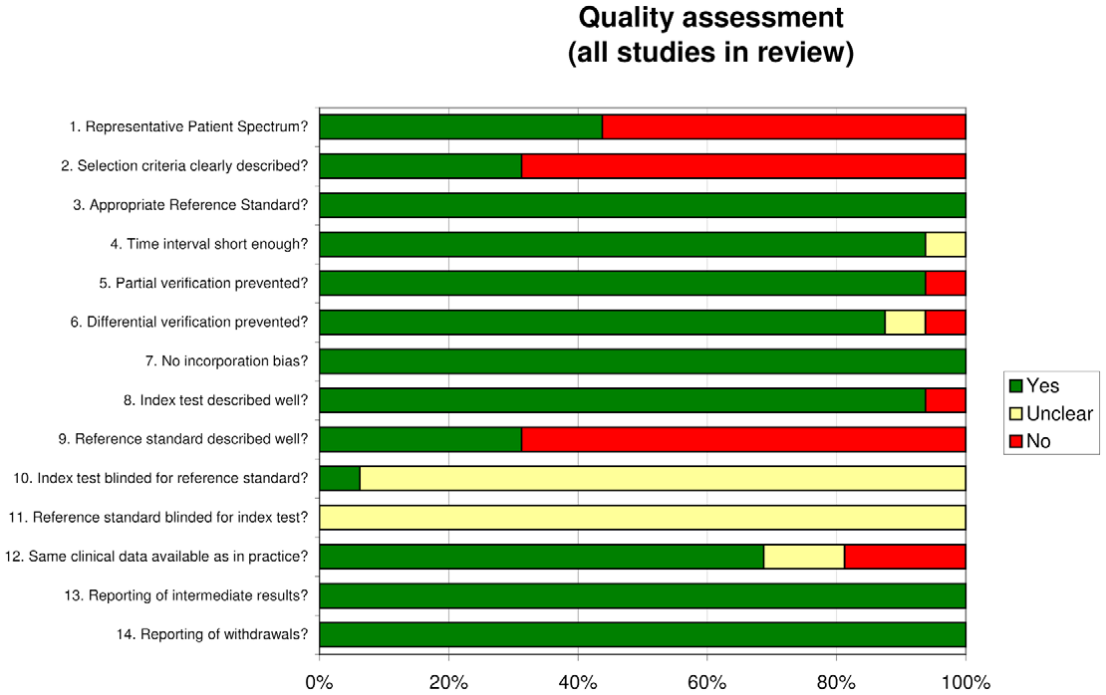
QUADAS results.

All articles were scored with the QUADAS tool (quality assessment for diagnostic accuracy) which included, amongst other, scoring based upon patient spectrum, blinding, exclusion and inclusion criteria (Figure 2). Studies performed badly when assessed for using representative patient populations. The majority of the studies seemed to enroll their patients in a consecutive way, although they did select them from highly skewed populations: in most articles, patients with confirmed HAT were enrolled, after which these patients underwent both the reference standard (microscopy) and the index test. This could artificially increase the clinical accuracy of tests. Only seven out of 16 articles included a representative patient spectrum, that is, patients suspected of infection with HAT.

In addition, all studies were scored ‘unclear’ when assessed for blinding of the reference standard to the index test results and vice versa (items 10 and 11 of QUADAS). There is a chance of bias if readers had prior knowledge of either the index or reference test outcome. The verification process (items 3 to 7 of QUADAS) raised no problems in most of the articles and the execution of the index test was sufficiently described (item 8) in all articles except one [30]. The aspect of withdrawals (item 14) was not applicable for most of the studies; 2 articles explained the withdrawal of patients from the study (Figure 2).

### Accuracy of molecular amplification tests for diagnosing HAT

**Figure 3:**
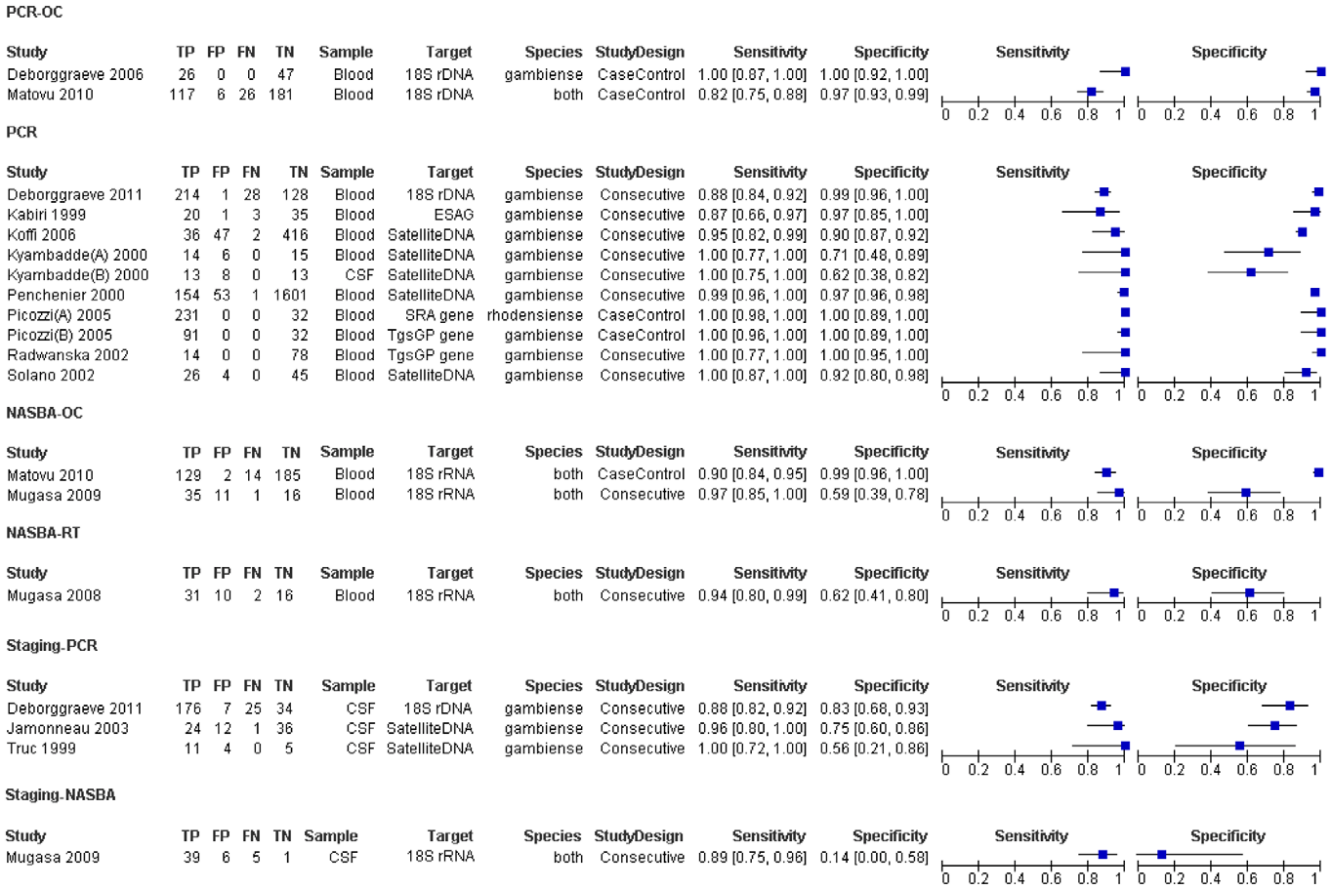
Forest plots. Overview of all 2 by 2 tables with forest plot (TP = true positives; FP = false positives; FN = false negatives; TN = true negatives; CSF = cerebrospinal fluid; PCR = polymerase chain reaction; NASBA = nucleic acid sequence based amplification; OC = oligochromatography; RT = real-time). Capital A or B refers to different set of data from the same paper. These sets may differ in clinical specimen studied, target gene or amplification technology applied.

**Figure 4:**
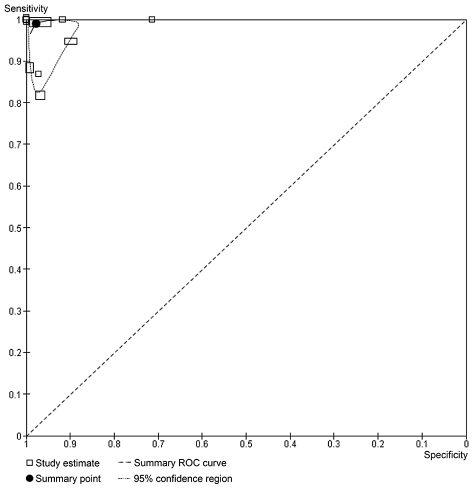
Raw ROC plot. Summary ROC plot for PCR and PCR-OC. Shows summary estimate (black dot), summary curve and confidence ellipse around the summary estimate. Width of the symbols reflects the number of non-diseased patients; height of the symbol reflects the number of diseased patients.

Two publications did not report sufficient data to construct the complete 2×2 tables, so these were excluded from the meta-analyses [13], [32]. The ten papers that reported on the accuracy of molecular tests for the diagnosis of HAT, included 15 separate studies and their respective, complete 2×2 tables. Their sensitivity varied from 82% to 100% and the specificity ranged from 59% to 100% (Figure 3). Eleven studies analysed PCR or PCR-OC in blood; their pooled sensitivity was 99.0% (95% CI 92.8 to 99.9%) and the pooled specificity was 97.7% (95% CI 93.0 to 99.3%) (Figure 4). There was no significant difference between the clinical accuracy of PCR and PCR-OC performed on blood samples (Table 2). Two studies assessed NASBA-OC, their sensitivities were 90.2% and 97.2%; their specificities were 98.9% and 59.3% respectively. The only study evaluating NASBA-RT in blood had a sensitivity of 93.9% and a specificity of 61.5%.

**Table 2:** Meta-analysis for PCR tests completed on blood, including subgroup analyses.

Main analysis	Studies (n)	Sensitivity (95% CI)	Specificity (95% CI)
PCR and PCR-OC combined	11	99.0% (92.8 to 99.9%)	97.7% (93.0 to 99.3%)

Subgroup analysis	Studies (n)	Sensitivity (95% CI)	Specificity (95% CI)
PCR	9	99.1% (93.8 to 99.9%)	97.4% (91.4 to 99.2%)
PCR-OC	2	96.5% (46.5 to 99.9%)	99.0% (86.7 to 99.9%)
p-value		0.471	0.506
PCR test on blood, satellite gene	5	99.1% (93.0 to 99.9%)	91.4% (82.6 to 96.0%)
PCR test on blood, other target genes	6	97.6% (85.7 to 99.6%)	99.5% (97.5 to 99.9%)
p-value		0.428	0.002
PCR test on blood, case-control studies	4	98.7% (82.9 to 99.9%)	99.8% (95.5 to 100%)
PCR test on blood, non-case-control studies	7	98.6% (90.7 to 99.8%)	94.5% (86.8 to 97.8%)
p-value		0.937	0.045

OC: Oligochromatography.

### Investigation of heterogeneity

The largest group of studies evaluated PCR (including PCR-OC) on blood. It was performed on five different targets: *T. gambiense* specific glycoprotein (TgsGP) [27], [31], serum resistance associated gene (SRA) [27], expression-site–associated genes 6 and 7 (ESAG 6/7) [11], 18S ribosomal DNA [23,23] and the satellite DNA [26], [28]–[30]. Target genes differ in copy number from TgsGP and SRA as single copy targets, ESAG with 10 copies, 18S rDNA with 40–200 copies and the satellite DNA with approximately 10,000 copies. We compared satellite sequences versus the other target sequences, which showed that using the satellite sequences as a target had a significantly lower specificity (p = 0.002, see Table 2).

Another source of heterogeneity is the infecting sub-species (*T.b. rhodesiense* or *T.b. gambiense*) as patients with *T.b.g* usually have a lower parasitaemia than patients with *T.b.r.* In addition, detection of the sub-species specific genes rather than the abundant genes that may appear in both sub-species also changes the diagnostic accuracy. Of the 11 PCR studies conducted on blood, one amplified *T. b. rhodesiense*-specific genes [27] and two amplified *T. b. gambiense*-specific genes [27], [31]. The remaining nine studies were species-specific amplifying *T. brucei s.l.*, thus amplifying the genes from both subspecies. The advantage of this method is that it is known to increase sensitivity. A separate analysis of the seven studies in patients infected with *T. b. gambiense*, using a PCR detecting both subspecies revealed a sensitivity of 97.6% (95% CI 90.8 to 99.4%) and a specificity of 95.8% (95% CI 88.9 to 98.5%).

### Study design

Of the 11 PCR studies on blood, six were diagnostic accuracy studies that enrolled consecutive suspects, the other five were case-control studies. The non case-control studies showed a pooled sensitivity of 98.6% (95% CI 90.7 to 99.8%) and a pooled specificity of 94.5% (95% CI 86.8 to 97.8%). In the case-control studies, the pooled specificity was significantly higher: 99.8% (95% CI 95.5 to 100%). The sensitivity did not significantly differ between the different types of study design: 98.7% (95% CI 82.9 to 99.9%). See also Table 2.

### Accuracy of molecular amplification tests for staging HAT

Four studies evaluated the accuracy of molecular tests to differentiate between stage one and stage two HAT. Three of these evaluated PCR in CSF while one evaluated NASBA-OC. The sensitivity of the PCR tests varied from 88% to 100%, while their specificity varied from 56% to 83%. The sensitivity of the NASBA-OC study was 88.6% and its specificity was 14.3%.

## Discussion

Molecular tests have been proposed as sensitive diagnostic tools for HAT; however, the accuracy of these tests for diagnosis has not yet been fully verified. In this systematic review, we analyzed the data from all available accuracy studies on molecular amplification tests for HAT, in order to better guide adoption of these tests in practice as possible triage, replacement or supportive diagnostic tests. From the available literature, conclusions can only be drawn about the accuracy of PCR tests for the diagnosis of HAT in blood. Overall, the 11 studies that analyzed PCR tests (both PCR and PCR-OC) on blood showed a high summary sensitivity of 99.0% and a specificity of 97.7%. Insufficient evidence was available about the accuracy of other molecular tests or about the ability of molecular tests to distinguish between stage I and stage II HAT.

More insight into the optimal place of PCR in practice and the types of PCR that can be used can be gained by the results of our subgroup-analyses. One source of variation that had a significant effect on diagnostic accuracy of PCR for HAT was the target DNA sequence of the test. Studies that used satellite DNA as target sequence showed significantly lower specificity than studies that used other target sequences. The satellite DNA target is highly specific and conserved among the *Trypanozoon* of which two subspecies of *Trypanosoma brucei* cause HAT [12]. The lower specificity may be due to contamination problems during DNA collection, extraction or amplification or inoculations with *T. b. brucei* which can circulate in blood of people with a regular challenge to tsetse fly bites [33]. It can also be caused by the design of the studies where we see more representative patient groups as compared with other studies. In addition, we do expect to see a high number of false positives if the reference test (here PCR on satellite DNA) is more sensitive than the index test.

Other factors that may have played a role could not be tested for their significance due to too few studies, such as sub-species detected and clinical sample used. The type of read-out system, (e.g. gel electrophoresis, OC) did not seem to affect accuracy. We found only one study analysing PCR diagnosis of *T. b. rhodesiense*. Accuracy results from *T. b. gambiense* can not necessarily be generalised for this sub-species and we recommend further diagnostic accuracy studies for *T. b. rhodesiense*. However, the parasitaemia for *T. b. rhodesiense* is generally higher than that of *T. b. gambiense* and disease progression is faster. We may, therefore, expect that molecular tools would have a high accuracy as more parasite DNA is present in blood samples [34]. Of all studies included, five, analyzed molecular tests in CSF; only one of these used CSF for primary diagnosis. Therefore, no firm conclusion can be drawn regarding the difference between blood and CSF for diagnosing HAT.

Three studies evaluated the ability of PCR to diagnose stage II HAT using CSF [22], [24], [25]. Routinely, staging is performed by microscopic examination of CSF samples that are obtained by lumbar puncture of confirmed HAT patients. The CSF is examined for presence of trypanosomes and elevated white blood cell count >5 cells/µl [28], [35]. The sensitivity of PCR to distinguish between stage I and stage II HAT ranged from 88% to100% and its specificity ranged from 56% to 83%. Although the number of false positives and false negatives in each study is variable and strong conclusions can not be made, the percentages of false positives is concerning, as these patients would be treated with a high risk treatment and may not have stage II HAT. Lumbar puncture remains inevitable as staging is paramount given that the different stages of HAT are managed using different drugs and is required for both molecular and microscopic staging of disease [34], [36]. The difficulty in diagnosing stage II HAT reiterates the need for prompt and accurate diagnosis of stage I HAT.

### Limitations

Our results suffer from two main limitations, one regarding the representativeness of the included patients and the other regarding the reference standard. Of the 11 studies in our main analysis (accuracy of PCR tests), only four included a representative patient spectrum. This may be a threat for the validity of the results shown here and for the translation of the results into practice. Diagnostic accuracy is not a fixed property of a test and may change over populations, especially when these populations are suffering from selection bias [19], [37], [38]. The most severe form of selection bias is using a case-control design in which the cases are confirmed and known cases and the controls are healthy people. Four out of eleven PCR studies were case-control studies and these showed a significantly higher specificity, which is expected as the included healthy controls are known to lead to an overestimation of accuracy [39], [40]. Future studies should think carefully about the patients to include and choose the patient spectrum most closely matching the situation as found in practice, otherwise health workers are forced to rely on accuracy data that are not representative. We recommend the inclusion of clinically or serologically suspected persons; e.g. persons living in endemic regions with enlarged lymph nodes, irregular fever, headaches or other neurological symptoms or positive in a serological test.

The other limitation of the studies that are presently available is that most use microscopy as the reference standard. Microscopy, itself, may have a relatively low sensitivity, although most of the studies we included used a form of centrifugation in order to increase sensitivity [34], [41]. However, the highly toxic treatment administered to HAT patients should only be given after demonstration of the parasites, and therefore, microscopy remains the accepted reference standard for HAT. For this review it means that sensitivity is the percentage of microscopy-positive patients with a positive molecular test and specificity is the percentage of microscopy-negative patients with a negative molecular test. In reality, it is possible that the index tests have correctly diagnosed patients who have been missed by microscopy due to its low sensitivity. In such cases the accuracy, and especially the specificity, of the index test is underestimated. However, in diagnostic studies, if there are any disagreements between the reference standard and the index test then it is assumed that the index test is incorrect. Therefore, in diagnostic accuracy study designs the index tests, by definition, can never be better than the reference standard. Other study designs or analytic techniques are needed to get more information about the relative accuracy of PCR versus microscopy. Examples may be latent class analyses, decision analyses or longitudinal studies using another reference standard to compare both PCR and microscopy with [42].

Even if the accuracy of PCR tests may be close to perfect, implementation of molecular diagnostic tests in the low and middle income countries that are most affected by HAT will be a difficult and arduous task. Role-out could be hampered by more practical issues; the time it may take before a diagnosis is made, the need for a cold-chain, continuous electricity or expertly-trained staff. Development of simple and more appropriate molecular tests, such as LAMP, that may show the same high accuracy in due course, may be a solution. For now, an important role for PCR in the control of HAT may be in screening samples from serologically positive patients collected from the field in a central reference laboratory; the high accuracy, shown here, would allow epidemics of HAT to be spotted early and treatment directed towards these specific areas. Longitudinal impact studies, feasibility studies and cost-effectiveness studies may be warranted to gain further information about the practical application of molecular diagnostics for HAT and their position within the diagnostic algorithm.

In conclusion, PCR tests seem to have an acceptably high specificity and sensitivity for diagnosis of stage I HAT. This conclusion is, however, based on microscopy as reference standard and a patient population that was not always representative. Future studies should, therefore, first and foremost include those patients for which PCR may become the test of choice. More certainty about the practical value of PCR tests for HAT diagnosis should come from non-accuracy design studies, like feasibility or cost-effectiveness studies.

## Supporting Information

Appendix S1 **Search terms in MEDLINE and Embase.**
(DOC)

Checklist S1 **Prisma 2009 checklist for systematic reviews and meta-analyses.**
(DOC)
