# Supplementary material for: Diagnostic Accuracy of Molecular Amplification Tests for Human African Trypanosomiasis—Systematic Review
Source: PLoS Negl Trop Dis. 2012 Jan 10;6(1):e1438. doi: 10.1371/journal.pntd.0001438 (PMC3254661; doi:10.1371/journal.pntd.0001438)
Supplement: Appendix S1 — Search terms in MEDLINE and Embase. (DOC) [file pntd.0001438.s001.doc]

**Appendix S1:** Search terms in MEDLINE and Embase.
